# Repeated Fractions of X-Radiation to the Breast Fat Pads of Mice Augment Activation of the Autotaxin-Lysophosphatidate-Inflammatory Cycle

**DOI:** 10.3390/cancers11111816

**Published:** 2019-11-19

**Authors:** Guanmin Meng, Melinda Wuest, Xiaoyun Tang, Jennifer Dufour, YuanYuan Zhao, Jonathan M. Curtis, Todd P. W. McMullen, David Murray, Frank Wuest, David N. Brindley

**Affiliations:** 1 Department of Biochemistry, University of Alberta, Edmonton, AB T6G 2S2, Canada; guanmin@ualberta.ca (G.M.); xtang2@ualberta.ca (X.T.); 2 Cancer Research Institute of Northern Alberta, University of Alberta, Edmonton, AB T6G 2S2, Canada; mwuest@ualberta.ca (M.W.); Todd.Mcmullen@albertahealthservices.ca (T.P.W.M.); David.Murray5@albertahealthservices.ca (D.M.); wuest@ualberta.ca (F.W.); 3 Department of Oncology, Division of Oncologic Imaging, University of Alberta, Edmonton, AB T6G 2R7, Canada; jdufour@ualberta.ca; 4 Department of Agricultural, Food and Nutritional Science, University of Alberta, 410 Agriculture/Forestry Centre, 3-60D South Academic Building, Edmonton, AB T6G 2P5, Canada; yz3@ualberta.ca (Y.Z.); jonathan.curtis@ualberta.ca (J.M.C.); 5 Department of Surgery, University of Alberta, Edmonton, AB T6G 2R7, Canada; 6 Department of Oncology, Division of Experimental Oncology, University of Alberta, Edmonton, AB T6G 2E1, Canada

**Keywords:** adiponectin, adipose tissue, breast cancer, chemokines, cytokines, radiotherapy

## Abstract

Breast cancer patients are usually treated with multiple fractions of radiotherapy (RT) to the whole breast after lumpectomy. We hypothesized that repeated fractions of RT would progressively activate the autotaxin–lysophosphatidate-inflammatory cycle. To test this, a normal breast fat pad and a fat pad containing a mouse 4T1 tumor were irradiated with X-rays using a small-animal “image-guided” RT platform. A single RT dose of 7.5 Gy and three daily doses of 7.5 Gy increased ATX activity and decreased plasma adiponectin concentrations. The concentrations of IL-6 and TNFα in plasma and of VEGF, G-CSF, CCL11 and CXCL10 in the irradiated fat pad were increased, but only after three fractions of RT. In 4T1 breast tumor-bearing mice, three fractions of 7.5 Gy augmented tumor-induced increases in plasma ATX activity and decreased adiponectin levels in the tumor-associated mammary fat pad. There were also increased expressions of multiple inflammatory mediators in the tumor-associated mammary fat pad and in tumors, which was accompanied by increased infiltration of CD45+ leukocytes into tumor-associated adipose tissue. This work provides novel evidence that increased ATX production is an early response to RT and that repeated fractions of RT activate the autotaxin–lysophosphatidate-inflammatory cycle. This wound healing response to RT-induced damage could decrease the efficacy of further fractions of RT.

## 1. Introduction

Radiotherapy (RT) is a mainstay of cancer treatment, but it sometimes fails to eliminate residual cancer cells and it can produce adverse side effects, such as fibrosis. RT “kills” cancer cells in part by damaging their DNA. This, together with cell debris and released proteins, causes inflammation, which in turn can enhance the immunologic elimination of cancer cells [1,2,3]. Although inflammation can be a key component of RT, we propose that persistent activation of the autotaxin (ATX)–lysophosphatidate (LPA)-inflammatory cycle becomes maladaptive.

Extracellular LPA is synthesized mainly by ATX and it signals through six G protein-coupled receptors. LPA signaling is terminated by lipid phosphate phosphatases (LPP1 and LPP3) [4]. In adults, ATX is secreted in response to inflammation [5,6] to facilitate wound healing [5]. LPA increases the innate immune response [7] and promotes lymphocyte extravasation and the conversion of monocytes to macrophages, which maintains immune homeostasis [8]. Inflammation resolves when the tissue is repaired and ATX secretion decreases [5]. When inflammation is not resolved, chronic activation of the ATX–LPA-inflammatory cycle becomes maladaptive [7,9] in conditions such as pulmonary fibrosis, cirrhosis, rheumatoid arthritis, inflammatory bowel disease and cancers [10,11]. Many inflammatory conditions are accompanied by fibrosis, and LPA drives fibrosis through LPA_1_ receptors [12,13,14,15,16,17,18,19,20,21,22,23]. This is why the ATX inhibitor, GLPG1690 [24], and an LPA_1_ receptor antagonist (BMS986020) [25], have attenuated idiopathic pulmonary fibrosis in Phase 2a clinical trials.

ATX activity is hijacked in cancers (wounds that do not heal) [26,27,28]. Inflammation and decreased acquired immune responses are “hallmarks” of cancer [10,28,29] and chronic LPA signaling enables cancer cells to evade the immune system [7,9]. LPA also increases VEGF production, which stimulates the angiogenesis needed for tumor growth [30]. ATX is secreted directly by melanoma, glioblastoma and thyroid cancer cells [6,31]. By contrast, breast cancer cells express little ATX [31,32,33,34]. Indeed, ATX activity in human and mouse 4T1 breast tumors is low compared to that in the adjacent adipose tissue [35]. Inflammatory cytokines produced by tumors increase ATX secretion by adjacent breast adipose tissue [5,35]. This establishes an inflammatory cycle, since LPA signaling stimulates the production of more inflammatory cytokines and cyclo-oxygenase-2 (COX-2). These changes promote the accumulation of inflammatory macrophages [7,9], which aggravate the inflammation. Bi-directional inflammatory signaling between breast tumors and adipose tissue has been confirmed by other groups [36,37].

About 40% of ATX in mice is produced by adipocytes, and this increases when being fed a high-fat human diet [38,39]. ATX production increases in obesity, especially when adipose tissue is inflamed [7,10]. Inflamed adipose tissue increases the co-morbidities of insulin resistance, diabetes, dyslipidemia, hypertension and atherosclerosis (metabolic syndrome). These conditions are characterized by low plasma adiponectin concentrations [40] and LPA decreases adiponectin secretion [38]. ATX could contribute to the association of obesity with ~30% of breast cancers [41,42]. Inhibiting ATX activity in breast cancer in mice decreased the concentrations of TNFα and G-CSF in plasma by ~10-fold [35]. The concentrations of various inflammatory cytokines/chemokines were also decreased in the fat pad adjacent to the 4T1 breast tumors along with a decreased number of CD45+ leukocytes in both the tumor and the adjacent adipose tissue [35].

Increasing the expressions of ATX or LPA_1-3_ receptors in mammary epithelial cells increased the development of breast cancer in mice [43]. Increased expressions of ATX and LPA_3_ receptors are associated with the aggressiveness of human breast carcinomas in women [44]. ATX concentrations correlate with invasiveness [10,45,46] and the ATX gene (*ENPP2*) is one of the 40–50 most up-regulated genes in metastatic tumors [47,48,49]. LPA signaling in several cancers is caused by increased ATX activity and decreased expressions of LPP1 and LPP3 [50], which degrade LPA and attenuate its ability to signal. Inhibiting ATX activity [34,35], or increasing LPP1 activity, decreases breast tumor growth and metastasis in mice [51,52]. The effects of low LPP1 expression in breast cancer cells are partly mediated by increased secretions of matrix metalloproteinases, which decrease the collagen contents of the tumors [53].

LPA also decreases the “killing” of breast cancer cells by Taxol [32], tamoxifen [54] and doxorubicin [55]. This depends partly on stimulation of LPA_1_ receptors, which activates the production of antioxidant proteins and multi-drug resistance transporters through the stabilization of Nrf2 [55]. This in turn protects cancer cells from damage caused by chemotherapy. ATX inhibition, therefore, increased the ability of doxorubicin to inhibit breast tumor growth and metastasis in mice [55].

About 60% of breast cancer patients receive breast-conserving surgery (lumpectomy) followed by RT, which typically involves ~16 daily fractions of 2.65 Gy to the whole post-surgical breast. A post-RT-induced cytokine surge occurs [56] and this produces fatigue in patients [57]. We previously showed that a single γ-ray exposure of between 0.25 and 5 Gy to rat and human adipose tissues in vitro activates LPA signaling by increasing the levels of ATX, LPA_1_ and LPA_2_ receptors; COX-2; and multiple inflammatory cytokines [58]. These responses were a consequence of RT-induced DNA damage, which activated ATM, ATR, PARP-1 and NF-κB [58]. Inhibiting each of these steps decreased the RT-induced activation of the ATX-LPA-inflammatory cycle. Higher radiation doses to intestinal cells triggered a similar signaling cascade [59].

Studies using human adipose tissue cultures [58] and intestinal cells [59] established the mechanisms that generated the ATX-LPA-inflammatory responses. However, studies in vitro do not take into account how cumulative damage and inflammation of adipose tissue caused by multiple fractions of RT, surgery or the proximity of breast tumor cells, affect the subsequent, RT-induced activation of the ATX-LPA-inflammatory cycle. To address these questions, which are more relevant to the clinical use of RT, we performed experiments with female mice in which we used a small-animal image-guided RT platform (SARRP). This allowed us to administer repeated treatment-planned RT to mouse breast tumors or fat pads while minimizing peripheral tissue damage, as is achieved clinically.

Our study indicates that one fraction of RT to a breast fat pad in mice initiated increased ATX production, resulting in increased ATX concentration in the plasma. There was relatively little effect of one fraction of RT on the concentrations of inflammatory cytokines/chemokines in plasma and irradiated fat pad. However, the cumulative effects of three fractions of RT produced marked increases the concentrations of several inflammatory cytokines/chemokines. This work provides new information about how multiple fractions of RT to breast tissue during the treatment of breast cancer result in activation of the ATX-LPA-inflammatory cycle and how this could affect the therapeutic outcomes from the RT.

## 2. Results

### 2.1. Multiple Fractions of RT Augment Inflammatory Cytokine Secretion from Breast Adipose Tissue

The purpose of these studies was to investigate how repeated fractions of RT to the breast, as would be used in RT for breast cancer, affect the ATX-LPA-inflammatory cycle. To provide a preclinical model, we used an image-guided small-animal radiation research platform (SARRP) to administer treatment-planned RT to mouse breast tumors and/or fat pads. This minimized peripheral normal tissue damage, as is achieved clinically. We initially performed a series of experiments to establish that five doses of 7.5 Gy was well tolerated with no significant loss of body weight. This regimen decreased the growth of 4T1 breast tumors by ~80% [60].

**Figure 1:**
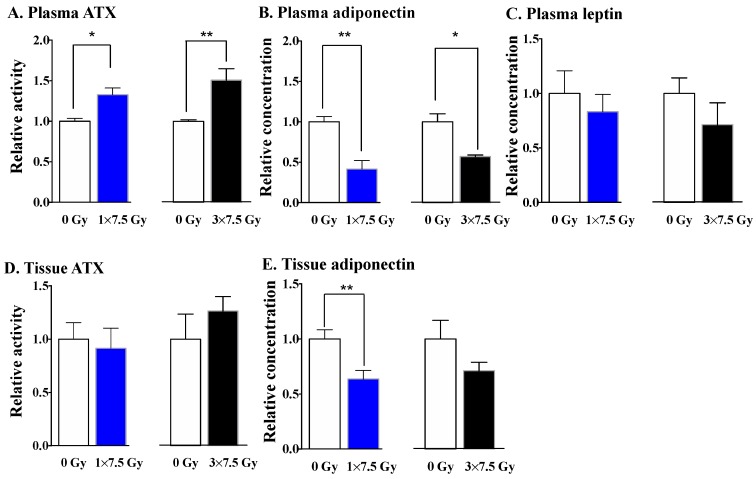
Comparing the effects of a single dose and three fractions of radiation therapy (RT) on ATX activity and the secretions of adiponectin and leptin in normal mice. Using the SARRP system, the 2nd left mammary fat pad of Balb/c mice received one fraction of 7.5 Gy (1 × 7.5 Gy), or three consecutive daily fractions of 7.5 Gy (3 × 7.5 Gy) of X-rays, or no irradiation (0 Gy). At 48 h after the completion of RT, the ATX activity in plasma (**A**) and irradiated fat pad (**D**); the levels of secreted adiponectin in plasma (**B**) and irradiated fat pad (**E**); and plasma leptin levels (**C**) were measured. Results are from 6–10 mice that were studied in two independent experiments. Results are expressed relative to tissue or plasma from non-irradiated mice as means ± SEMs. * *p* < 0.05 and ** *p* < 0.01. The absolute values were similar to those in our previous publication [61].

For the present work, we irradiated the 2nd left fat pad of normal (i.e., non-tumor-bearing) mice with a single dose of 7.5 Gy and compared this to three consecutive daily fractions of 7.5 Gy. Plasma and the irradiated fat pad were collected at 48 h after the final dose of RT. The single dose of RT increased plasma ATX activity by ~1.3-fold at 48 h post-RT, and decreased plasma adiponectin concentrations by ~58% compared to the control (Figure 1A,B). These changes in the plasma are remarkable since the RT was directed to a single mammary fad pad. A similar response was observed after treatment with three daily fractions of 7.5 Gy, which produced an ~1.5-fold elevation in plasma ATX activity, as well as an ~50% decrease in plasma adiponectin concentrations (Figure 1A,B). Neither of the RT regimens changed the leptin concentrations in plasma significantly, although there was a trend toward decreased levels in both cases (Figure 1C).

Three fractions of RT to the fat pad appeared to increase ATX activity in the irradiated fat pad, but this did not reach statistical significance (*p* = 0.086) (Figure 1D). The single dose of RT decreased the adiponectin concentration in the irradiated fat pad; the effect of the three-dose RT regimen showed the same trend, although the results did not reach statistical significance (*p* = 0.149) (Figure 1E).

**Figure 2:**
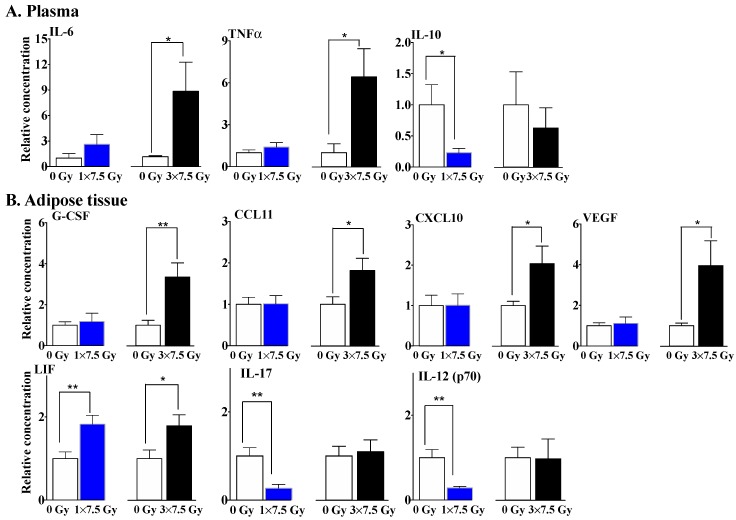
Three fractions of RT increased the concentrations of cytokines, chemokines and growth factors in plasma and in the irradiated breast adipose tissue of normal mice compared to a single dose of RT. The 2nd left mammary fat pad of Balb/c mice was exposed to single dose of 7.5 Gy of X rays (1 × 7.5 Gy) or three fractions of 7.5 Gy (3 × 7.5 Gy), and the plasma and irradiated fat pad were collected for multiplex cytokine analyses 48 h after the completion of RT. Results of the secretions of cytokines and chemokines in the plasma (**A**) and irradiated fat pad (**B**) are expressed relative to tissue or plasma from non-irradiated mice as means ± SEMs from 6–10 mice from two independent experiments. * *p* < 0.05 and ** *p* < 0.01. These values were similar to those in our previous publication [61].

The differential impact of multiple doses of RT was more apparent in plasma and mammary adipose tissue when comparing some cytokines, chemokines and growth factors, which play essential roles in controlling inflammation and immune responses. There was relatively little effect of a single dose of RT on the plasma concentrations of the pro-inflammatory cytokines, IL-6 and TNFα, whereas three fractions of RT elevated their expressions by 8.84 and 6.43-fold, respectively (Figure 2A). Conversely, one fraction of RT decreased the plasma concentration of the anti-inflammatory cytokine IL-10 by ~80%, although the decrease was not statistically significant after three fractions of RT.

In the mammary adipose tissue, there were no significant changes in G-CSF, CCL11, CXCL10 or VEGF after the single dose of RT, whereas these concentrations were increased by 3.35, 1.82, 2.04 and 3.95-fold after three fractions of RT, respectively (Figure 2B). By contrast, three fractions of RT had no significant effect on the IL-17 and IL-12 (p70 subunit) levels in adipose tissue, whereas a single fraction of RT decreased their expression levels by ~80% (Figure 2B). Both the single and three-fraction RT regimens produced an increase of ~1.8-fold in the expression of leukemia inhibitor factor (LIF) in adipose tissue.

Taken together, the increased ATX activity and decreased adiponectin secretion show similar responses to the different RT regimens, suggesting their potential roles as early markers of RT-induced inflammation. By contrast, several cytokines and chemokines show much higher concentrations after three fractions of RT, suggesting an augmented inflammatory milieu caused by the accumulated tissue damage resulting from repeated RT delivery.

**Figure 3:**
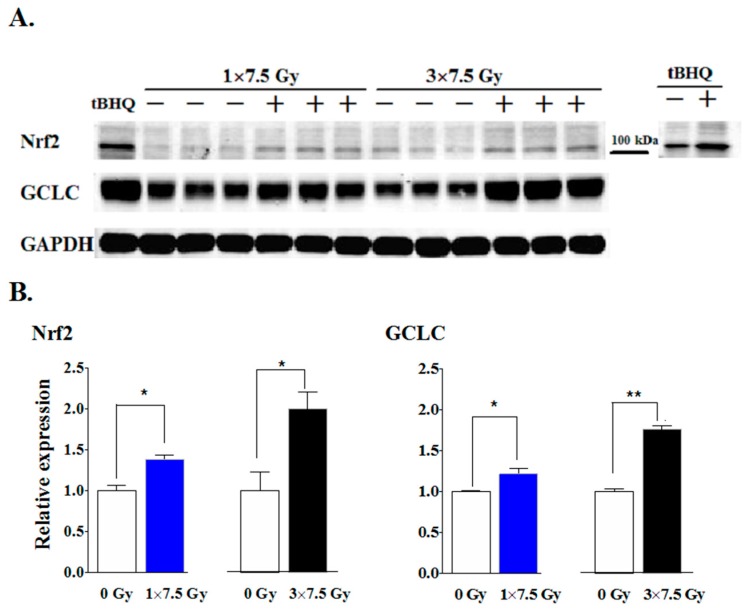
One or three fractions of RT enhanced the levels of Nrf2 and GCLC in adipose tissue of normal mice. (**A**) The 2nd left mammary fat pad of mice was exposed to a single dose of RT (1 × 7.5 Gy) or three fractions of RT (3 × 7.5 Gy). Mammary adipose tissue was collected and the levels of Nrf2 and GCLC were determined by western blot analysis 48 h after the completion of RT. Cultured 4T1 cells were treated with 10 μM tBHQ for 6 h as a positive verification for Nrf2 induction. (**B**) The protein levels of Nrf2 and GCLC were expressed relative to non-irradiated mice and normalized to glyceraldehyde phosphate dehydrogenase (GAPDH). Results are means ± SEMs from five mice. * *p* < 0.05 and ** *p* < 0.01. The complete western blot is shown in Supplementary Figure S1.

Next, we examined the effects of the single and three-dose RT regimens on the nuclear factor erythroid 2-like factor 2 (Nrf2) transcription factor, a well-known master regulator for maintaining redox homeostasis [62]. Nrf2 activation increases the expression of many enzymes involved in the removal of potentially damaging oxidative and electrophilic species and in DNA repair [63,64,65]. Notably, a single fraction of RT increased the levels of Nrf2 and its downstream target protein, glutamate-cysteine ligase catalytic subunit (GCLC), in adipose tissue at 48 h post-irradiation (Figure 3A,B). These effects were greater 48 h after completion of the three-fraction RT regimen. The specificity of the Nrf2 antibody was verified (Figure 3A) by treatment of 4T1 cells with tBHQ, a known Nrf2 activator, as in previous work [55,66]. These results show that the activation of Nrf2 and downstream antioxidant response element (ARE)-regulated genes were enhanced by accumulated RT-induced damage.

**Figure 4:**
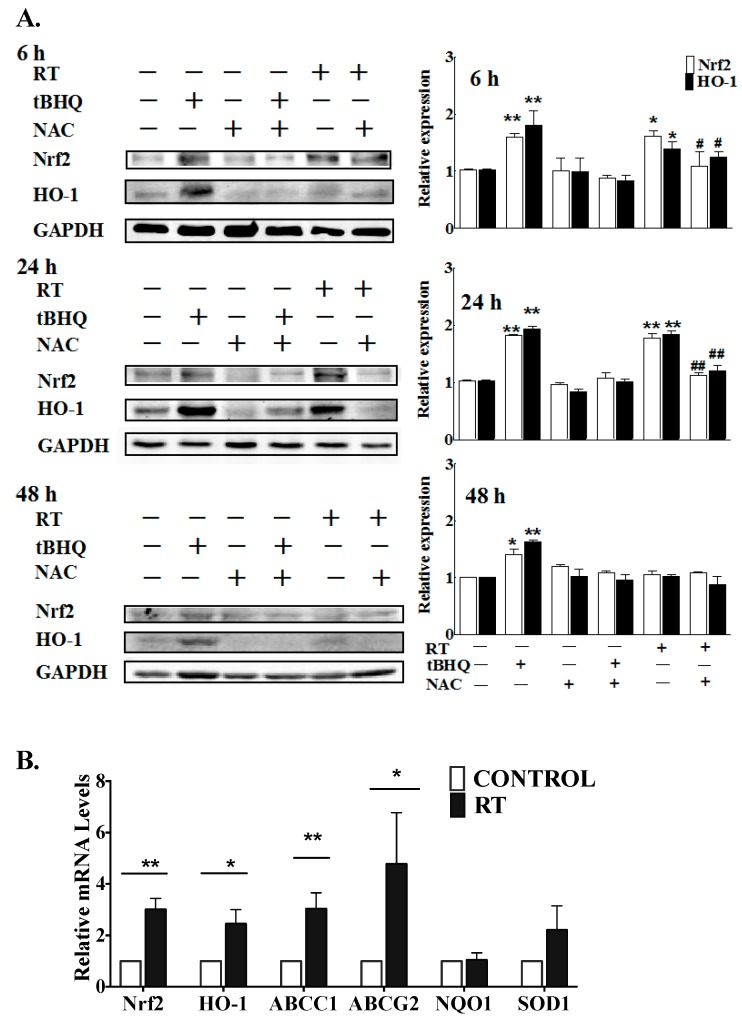
RT-activated Nrf2 and its down-stream targets after 6 h to 24 h in human adipose tissue. (**A**) Human adipose tissue samples were treated with tBHQ (50 µM), with or without NAC (20 µM), for 3 h, or were exposed to 1 Gy of γ-radiation either with or without a 3 h pretreatment with NAC (20 µM), as indicated. Levels of Nrf2 and HO-1 were determined by western blot analysis at various time points after irradiation (6, 24 and 48 h). The results (right-hand panels) were quantified relative to the non-irradiated control. Results are given as means ± SEMs from four independent experiments. * *p* < 0.05; ** *p* < 0.01 versus control; # *p* < 0.05; ## *p* < 0.01 versus radiation without NAC. (**B**) Cultured human adipose tissue was exposed to 1 Gy of γ-radiation and mRNA expression of Nrf2 and of five known Nrf2/ARE-regulated genes were measured after 24 h. Results are expressed as means ± SEMs from five independent experiments. * *p* < 0.05 and ** *p* < 0.01 versus control. The complete western blot is shown in Supplementary Figure S1.

To further substantiate this work, we examined the effects of a single 1 Gy γ-ray exposure on cultured human adipose tissue. This caused a significant increase in the levels of the Nrf2 protein during the period from 6 h to 24 h, and of the downstream effector heme oxygenase-1 (HO-1) protein, an antioxidant enzyme, with both of these increases declining by 48 h after the completion of RT (Figure 4A). The inductions of Nrf2 and HO-1 were effectively reversed by pretreatment with the thiol antioxidant, N-acetylcysteine (NAC). The mRNA expressions of several Nrf2 downstream target genes was also elevated at 24 h after a 1 Gy γ-ray exposure, including *HO-1*, *ABCC1* and *ABCG2* (Figure 4B).

### 2.2. The Effects of Three Fractions of RT in Tumor-Bearing and Normal Mice

**Figure 5:**
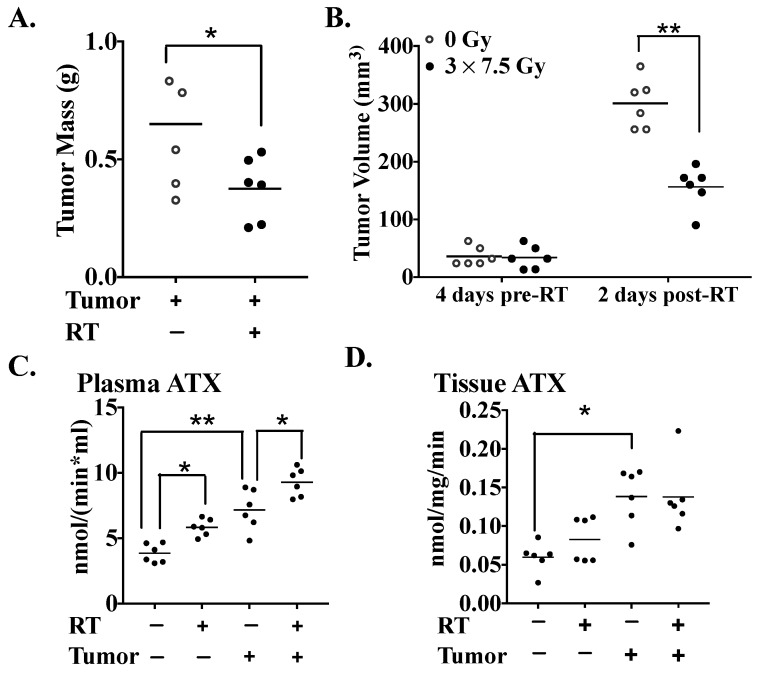
Three fractions of RT reduced tumor growth and augmented the tumor-induced increases in plasma ATX activity for tumor-bearing and normal mice. Both 4T1 tumor-bearing mice and normal mice were either untreated or were irradiated daily with 7.5 Gy of X-rays for three successive days, beginning on day 12 after tumor-cell injection or lack thereof. (**A**) Mass of tumors excised 48 h after completion of RT. (**B**) Tumor volumes four days before the start of RT and at 48 h after completing the RT. At 48 h after the completion of RT, the ATX activity in both plasma (**C**) and the mammary fat pad (**D**) were measured. Results are expressed as means ± SEMs for six mice/group. * *p* < 0.05 and ** *p* < 0.01.

Having established that three fractions of RT produces a greater increase in inflammatory cytokines and chemokines compared to one RT fraction, we next investigated how adipose tissue that was already inflamed would respond to three fractions of RT. To produce this inflammation, we used mice where the adipose tissue was inflamed by the presence of a breast tumor. Balb/c mice were injected into the 2nd left mammary fat pad with syngeneic 4T1 breast cancer cells, and the first RT treatment was delivered when the tumors had been growing for 12 days. Three daily 7.5 Gy fractions of RT to the tumor and associated fat pad decreased tumor weight by ~43% (Figure 5A) and tumor volume by ~50% (Figure 5B) by the time the mice were killed—48 h after completing the RT. Tumor-bearing mice showed increased plasma ATX activity compared to normal mice, as expected (Figure 5C) [34]. Notably, three fractions of RT increased plasma ATX activity in normal and tumor-bearing mice by a further ~30%. The breast fat pad tissue associated with the tumor showed higher ATX activity than the fat pad of normal, non-irradiated mice, but this ATX activity was surprisingly not significantly increased by RT (Figure 5D).

**Figure 6:**
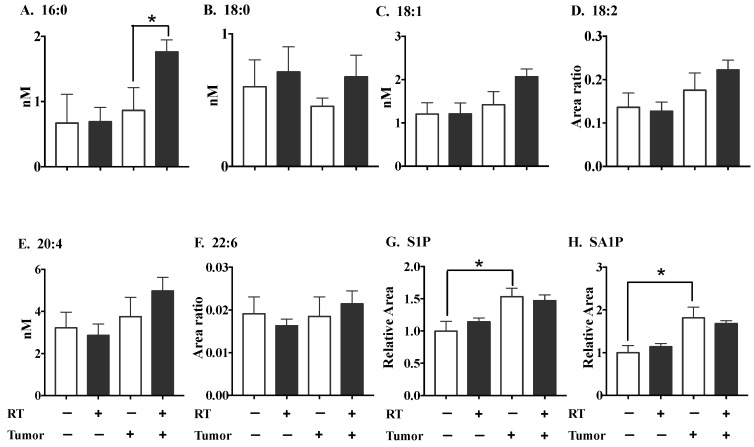
The effect of three 7.5 Gy fractions of RT on plasma concentrations of LPA species, S1P and SA1P in tumor-bearing mice and normal mice. Mice with or without breast tumors were either untreated or were treated daily with X-rays for 3 days, and the plasma concentrations of LPA species (**A**–**F**), S1P (**G**) and SA1P (**H**) were measured 48 h after the completion of RT. Absolute amounts of C16:0-LPA, C18:0-LPA, C18:1-LPA and C20:4-LPA were determined from the calibration curves, while LPA-18:2 LPA-22:6, S1P and SA1P were calculated from the peak areas relative to the isotopically-labelled standards. Results are expressed as means ± SEMs with six mice/group. * *p* < 0.05 and ** *p* < 0.01.

We determined next whether the RT-induced increase in ATX activity would be reflected in plasma LPA concentrations in the 4T1 breast tumor model. Mice with breast tumors showed higher circulating LPA-16:0 levels after three fractions of RT compared to non-irradiated, tumor-bearing mice (Figure 6). However, three fractions of RT had no significant effect on the plasma concentrations of LPA-18:0, LPA-18:1, LPA-18:2, LPA-20:4, LPA-22:6, sphingosine 1-phosphate (S1P) and sphinganine 1-phosphate (SA1P) in either normal or tumor-bearing mice. The levels of S1P and SA1P (but not of any of the LPA species) were increased by the presence of a tumor. Three fractions of RT did not alter the LPA concentrations in the tumors (Supplementary Figure S2).

**Figure 7:**
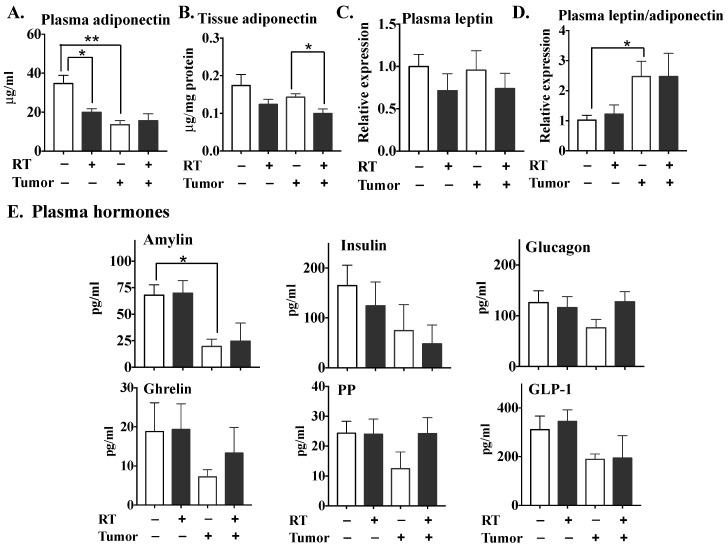
The effects of three 7.5 Gy fractions of RT on the levels of adiponectin, leptin and various hormones in tumor-bearing and normal mice. At 48 h after completing the RT, the secretions of adiponectin in plasma (**A**) and irradiated fat pads (**B**), and plasma leptin concentrations (**C**), were measured, and the leptin/adiponectin ratio (**D**) was calculated accordingly. (**E**) Concentrations of hormones including amylin, insulin, glucagon, ghrelin, PP and GLP-1 were analyzed by mouse metabolic multiplex arrays. Results are expressed as means ± SEMs with six mice/group. * *p* < 0.05 and ** *p* < 0.01.

Adiponectin secretion by adipose tissue exhibits anti-inflammatory activity and is inversely linked to the risk of obesity-associated malignancies and insulin resistance [67]. Plasma adiponectin levels were decreased significantly by three fractions of RT in normal mice (Figure 7A). Plasma adiponectin was significantly lower in tumor-bearing mice and this level was not decreased further by RT. Adipose tissue associated with the tumor exhibited lower adiponectin concentrations than normal adipose tissue, although this effect did not reach statistical significance. Three fractions of RT appeared to decrease adiponectin levels in both normal and tumor-associated adipose tissue, although the effect only reached statistical significance in the latter instance (Figure 7B). RT did not change the adiponectin concentrations in the tumor itself (results not shown). Plasma leptin levels were not altered significantly by the presence of the tumor or by RT (Figure 7C). Although the leptin/adiponectin ratio was increased in tumor-bearing mice, RT had no effect on this ratio in either tumor-bearing or normal mice (Figure 7D).

Interestingly, tumor-bearing mice showed substantially decreased plasma levels of amylin (Figure 7E), which is co-secreted with insulin by pancreatic β–cells. However, these levels were not affected significantly by RT in the control or normal mice. The plasma concentrations of hormones which maintain glucose homeostasis and regulation of body weight, including insulin, glucagon, glucagon-like peptide 1 (GLP-1), ghrelin and pancreatic polypeptide (PP), were generally but not significantly lower in tumor-bearing versus normal mice and were not altered significantly by RT in either case.

**Figure 8:**
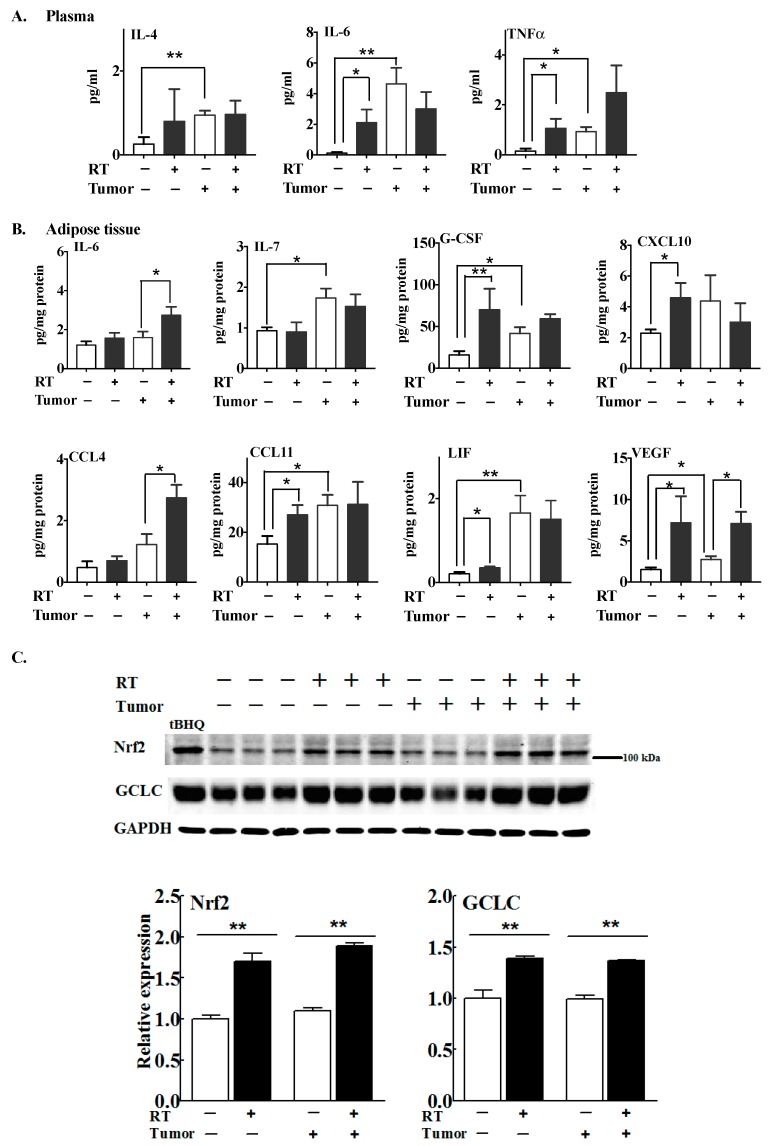
The effect of three 7.5 Gy fractions of RT on the concentrations of some cytokines, chemokines and growth factors in tumor-bearing and normal mice. The concentrations of cytokines, chemokines and growth factors in plasma and in adipose tissue were analyzed by mouse cytokine/chemokine multiplex arrays 48 h after the completion of RT. The levels of cytokines and chemokines in the plasma (**A**) and adipose tissue (**B**) are expressed as means ± SEMs from six mice/group. * *p* < 0.05 and ** *p* < 0.01. (**C**) The protein levels of Nrf2 and GCLC in mammary adipose tissue were expressed relative to the control from normal (i.e., non-tumor-bearing) mice that were not irradiated. Results are means ± SEMs from six mice/group. * *p* < 0.05 and ** *p* < 0.01. The complete western blot is shown in Supplementary Figure S1.

Plasma IL-4, IL-6 and TNFα levels were significantly increased in tumor-bearing versus normal mice (Figure 8A). Three fractions of RT also significantly increased the plasma concentrations of IL-6 and TNFα in the normal mice, but not in the tumor-bearing mice. The concentrations of five cytokines out of 32 compounds analyzed on the array—IL-7, G-CSF, CCL11, LIF and VEGF—were higher in the tumor-associated adipose tissue than in normal mouse adipose tissue (Figure 8B). Three fractions of RT elevated the concentrations of G-CSF, CXCL10, CCL11, LIF and VEGF in the adipose tissue of normal mice. In the tumor-bearing mice, IL-6, CCL4 and VEGF were increased by RT in the tumor-associated fat pad.

Elevation of Nrf2 by RT might be an important factor in regulating the response of adipose tissue to RT (Figure 3). The levels of Nrf2 and its downstream target, GCLC, in the mammary fat pad, were not influenced by the presence of a tumor. The increases in Nrf2 and GCLC following RT were similar in normal and tumor-associated adipose tissue (Figure 8C).

**Figure 9:**
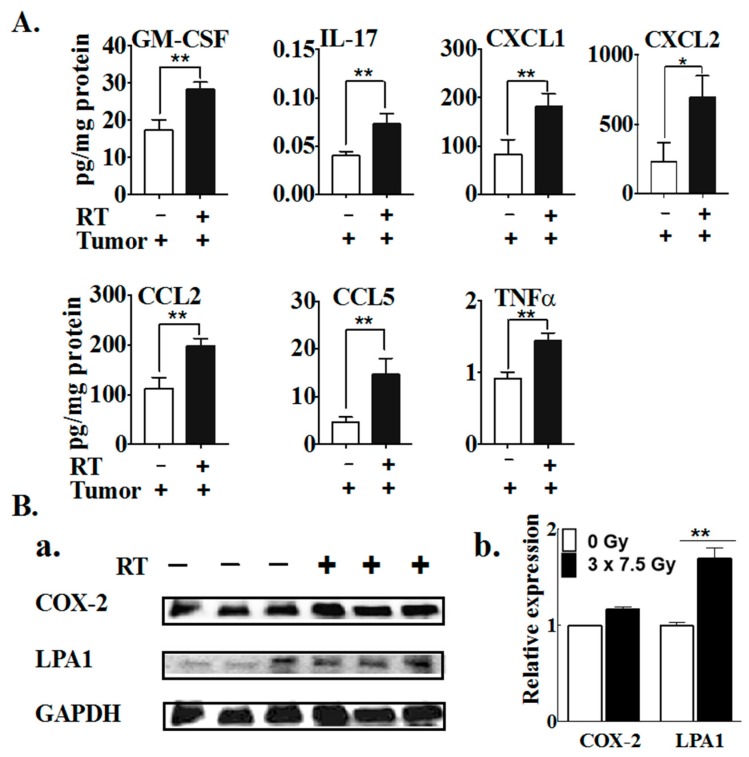
Three 7.5 Gy fractions of RT elevated the secretions of cytokines and chemokines, and increased the expression of the LPA1 receptor in tumors. (**A**) The concentrations of cytokines, chemokines and growth factors in the tumor tissue were analyzed by mouse cytokine/chemokine multiplex array 48 h after the completion of RT. (**B**) The protein levels of COX-2 and LPA_1_ receptors were determined by western blot analysis in the tumor tissue and the relative expressions were normalized to the level of GAPDH. Results are expressed as means ± SEMs with six mice/group. * *p* < 0.05 and ** *p* < 0.01 versus tumor alone. The complete western blot is shown in Supplementary Figure S1.

Three fractions of RT increased the concentrations of seven inflammatory mediators in tumor tissues: GM-CSF, IL-17, CXCL1, CXCL2, CCL2, CCL5 and TNFα (Figure 9A). RT markedly increased LPA_1_ receptor levels in tumors, but did not alter COX-2 expression significantly (Figure 9B).

**Figure 10:**
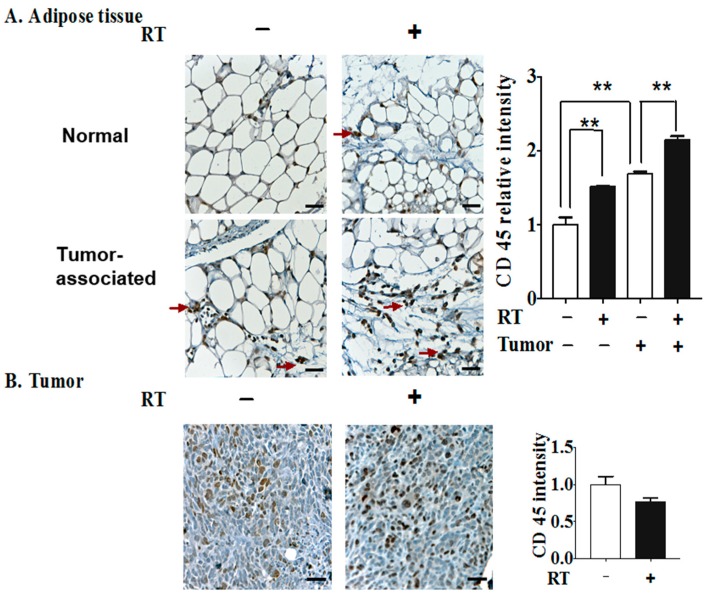
Three 7.5 Gy fractions of RT enhanced the infiltration of inflammatory cells into tumor-associated adipose tissue 48 h after the completion of RT. (**A**) Representative images of leukocyte/macrophage infiltration indicated by CD45 staining (red arrows) in the mammary fat pad from normal (i.e., non-tumor-bearing) mice or in the mammary fat pad adjacent to a 4T1 breast tumor (tumor-associated). (**B**) CD45+ leukocytes within tumor tissues from irradiated or non-irradiated mice. The quantifications of CD45 staining are shown in the right-hand panels for six mice. ** *p* < 0.01. Scale bar = 50 μm.

We next studied if inflammation caused by the tumor or RT would be reflected in the accumulation of leukocytes using CD45 as a marker. The numbers of CD45+ cells in the tumor-associated mammary fat pad were increased relative to normal adipose tissue (Figure 10A). The infiltration of CD45+ leukocytes into both normal and tumor-associated adipose tissue was increased by RT, which is compatible with the previous finding that leukocytes, including macrophages, are a major contributing source of inflammatory cytokines in adipose tissue in addition to adipocytes themselves [68]. However, the levels of CD45+ leukocytes/field in tumor tissue were not altered significantly by RT (Figure 10B).

## 3. Discussion

About 60% of breast cancer patients are treated with breast conserving therapy (lumpectomy), followed typically by multiple daily fractions of either 1.8-2.0 (conventional) or ~2.65 Gy (hypofractionation) to the whole post-operative breast to eliminate residual cancer cells. As a result, adipocytes in the irradiated breast suffer repeated DNA damage, resulting in the activation of ATM, ATR, PARP-1 and NF-κB. Adipocytes are a major site of ATX secretion in adipose tissue and their DNA damage caused by RT increases ATX secretion [58]. This effect occurs after irradiation with 0.5 to 5 Gy, which encompasses the therapeutic range. Increased LPA production from ATX aggravates inflammation by increasing the expressions of COX-2 and multiple inflammatory cytokines, which induces further ATX secretion in a feed forward cycle [58]. Inflammation is an important component of RT since it may enhance the immune elimination of cancer cells [1,2,3]. However, the ATX-LPA-inflammatory cycle is part of a wound healing response that can potentially protect cells from RT-induced cell death [59,69,70,71]. The inflammatory milieu that is created by RT also promotes fibrosis, which is a common long-term morbidity associated with RT.

In the present work, we extended our previous studies [58] where we irradiated cultured human adipose tissue with a single dose of 0.25 to 5.0 Gy to study the effects of fractionated treatment with RT in an in-vivo mouse model of breast cancer. This enabled us to evaluate the impact of accumulated damage from RT such as would occur during breast cancer treatment. To do this, we used a SARRP system to administer targeted RT to a breast tumor or to a breast fat pad that did not contain a tumor. Remarkably, irradiating a single breast fat pad once or three times increased the plasma activity of ATX. This was accompanied by an increase in the plasma concentrations of 16:0-LPA in tumor-bearing mice. The concentrations of plasma S1P, which is the sphingolipid analogue of LPA, were increased in tumor-bearing mice. S1P also promotes inflammation, angiogenesis and cancer progression such that S1P receptors are potential therapeutic targets for cancer treatments [72,73,74,75].

RT and the presence of a breast tumor decreased the plasma adiponectin concentrations, which indicates an adverse effect of metabolic regulation [76]. The consequences of repetitive doses of RT were most obvious in cytokine responses. Three daily fractions of 7.5 Gy to the normal breast fat pad increased plasma concentrations of IL-6 and TNFα, whereas the concentration of IL-10, which is anti-inflammatory, was decreased. Multiple, but not single, fractions of RT also increased the concentrations in G-CSF, CCL11, CXCL10 and VEGF in the irradiated fat pad in normal mice. By contrast, three fractions of RT had no significant effect on the IL-17 and IL-12 (p70 subunit) levels in adipose tissue, whereas a single fraction of RT decreased their expressions. In another case, notably LIF, both single and three-fraction RT regimens increased its expression in adipose tissue. By contrast, one fraction of RT had little, if any, effect on the concentrations of inflammatory cytokines/chemokines in plasma and adipose tissue.

These effects demonstrate that the increase in ATX activity occurred after the first fraction of RT and that the increases in the concentrations of several cytokines/chemokines occurred later after multiple fractions of RT. The cumulative damage to DNA and tissues and the consequent increase in LPA signaling probably results in increased cytokine/chemokine production as part of a “wound healing response.” Similar to our observations, Desai et al. observed higher levels of secreted cytokines after 3 × 2 Gy compared to a single 2-Gy fraction in various human tumor cell lines [77].

In previous work, we showed that irradiating rat and human adipose tissue in culture with a single dose of 0.5 to 5.0 Gy increased the production of ATX. There was also an increase of ~50–150% in the concentrations of 14 cytokines/chemokines in the culture medium of irradiated human adipose tissue after exposure to 1 Gy of radiation [58]. These results contrast with the present experiments, which were conducted in vivo by exposing a mouse fat pad once or three times to 7.5 Gy of X-rays. One fraction of RT increased plasma ATX activity, but the apparent increases in the plasma concentrations of IL-6 and TNFα did not reach statistical significance. By contrast, these latter concentrations were increased by six to 9-fold after three daily doses of RT. Similarly, there were significant increases in the concentrations of G-CSF, CCL11, CXCL10 and VEGF in adipose tissue after three factions of RT, whereas one fraction had no significant effect. These results in vivo are likely to be more physiologically meaningful since they are steady-state values that reflect the rates of synthesis and removal of the compounds in question, as well as the inflammation that accrues from the observed leukocyte infiltration of the adipose tissue

The relationship between RT, the inflammatory microenvironment of the tumor and immune responses is complex [78,79]. The present experiments enabled us to study part of this complexity and interactions of breast tissue damaged by RT in vivo, which is not possible with cultured adipose tissue or cells. Although immune activation can be an integral part of the therapeutic effect of RT, fractionated RT can sometimes be immunosuppressive [80]. Tumor response to RT is not only influenced by the tumor type and tissue distribution, but also by the tumor inflammatory microenvironment. Elevated levels of inflammatory cytokines in a variety of tumors, e.g., IL-6 [81], IL-1β [82], TGF-β [83] and CXCL1 [84], have shown positive correlations with radioresistance in cancer cells, whereas blocking the effects of these cytokines/chemokines can enhance sensitivity to RT. Equally, increased ATX and LPA levels are known to protect cells from RT-induced damage [69].

We previously demonstrated that stimulation of LPA_1_ receptors by LPA activates the anti-oxidant response element through stabilization of Nrf2 [55]. This protects cancer cells from cell death caused by various anti-cancer treatments [55,85]. Constitutive Nrf2 activation promotes the resistance of cancer cells to RT [86]. In addition, Nrf2 has been reported to cross-talk with other radiation-responsive pathways. ATM regulates Nrf2 stability via protein kinase C delta (PKCδ) in the oxidative stress response [87]. Furthermore, various oxidants stabilize and activate Nrf2 through p21^WAF1^ induction [88], which, in the case of ionizing radiation, would be mediated largely by the ATM–p53 axis [65]. In human adipose tissue, we showed previously that ATM is activated by RT-induced DNA damage along with ATR, PARP-1 and NF-κB. This increases ATX secretion [58]. It is, therefore, possible that the stabilization of Nrf2 through locally produced LPA and activation of LPA_1_ receptors [55] could contribute to radioresistance by protecting against RT-induced oxidative damage and by increasing DNA repair. Although these effects could augment the effects of LPA through LPA_2_ receptors, which decrease the expression of Siva, a pro-apoptotic protein [69], apoptosis is not a major primary consequence of RT in breast cancer. Instead, human, solid tumor-derived cell lines, including breast cancer cells, typically undergo some form of cytostasis (senescence or polyploid giant-cell formation) after RT [89].

Surprisingly, we observed an increase in Nrf2 mRNA levels in cultured human adipose tissue after exposure to 1 Gy of γ-rays (Figure 4B) since changes in Nrf2 levels following oxidative stress are usually caused by modulation of its degradation [65]. However, it has been reported that Nrf2 transcription could be modulated by several anti-oxidant or anti-carcinogenic agents, such as Quercetin [90] and 3*H*-1,2-dithiole-3-thione (D3T) [91].

We hypothesized here that diminishing RT-induced activation of the ATX-LPA-inflammatory cycle would improve outcomes from RT. This is consistent with observations that ATX inhibition with BrP-LPA (also a pan-LPA receptor antagonist) or PF-8380 increased the sensitivity of heterotopic glioblastomas to RT in mice [92,93]. These compounds are not suitable for clinical use, but the ATX inhibitor GLPG1690 attenuated the progression of idiopathic pulmonary fibrosis in Phase 2 clinical trials [23,24]. Five fractions of 7.5 Gy of X-rays delivered using the SARRP system produced an ~80% decrease in the weight of 4T1 tumors [60]. Although GLPG1690 did not decrease tumor weight further compared to RT, it did decrease the uptake of 3′-deoxy-3′-[^18^F]-fluorothymidine, which occurs mainly in the cancer cells within the tumors [60]. There was also a GLPG1690-induced decrease in the percentage of Ki67 positive cells, indicating decreased proliferation of the cancer cells. These effects are compatible with RT-induced cytostasis. These combined results show that blocking the effects of RT-induced ATX expression provides a potential strategy for increasing the efficacy of RT.

The present work is the first to describe the effects of delivering treatment-planned RT to mouse breast fat pads and breast tumors and the activation of the ATX-LPA-inflammatory cycle. This experimental mouse model was used to mimic the administration of multiple fractions of RT to the breasts of cancer patients. RT causes damage to adipocytes, which are the major source of ATX in the body. Consequently, the increased secretion of ATX and signaling by LPA initiates a feedforward inflammatory cycle, which becomes greater with repeated fractions of RT. This inflammatory cycle can become maladaptive by producing an LPA-driven wound healing response than can potentially protect cancer cells from RT-induced damage and initiate RT-induced fibrosis.

## 4. Materials and Methods

### 4.1. Reagents

Penicillin/streptomycin and Roswell Park Memorial Institute (RPMI) 1640 medium were from Gibco/Life Technologies (Burlington, ON, Canada). Amplex^TM^ Red was from Invitrogen Life Technologies (Camarillo, CA, USA). The mouse adiponectin ELISA kit was from Cyrstal Chem Inc. (Elk Grove Village, IL, USA). Antibodies against LPA_1_ and CD45 were from Abcam (Cambridge, UK), and COX-2 antibody was from Santa Cruz Biotechnology, Inc. (Santa Cruz, CA, USA). HRP conjugated anti-rabbit IgG antibody was from DAKO (Carpinteria, CA, USA). All primers were purchased from Integrated DNA Technologies Inc. (Coralville, IA, USA). Other chemicals and reagents were from Sigma-Aldrich (St. Louis, MO, USA) unless indicated otherwise.

### 4.2. Mouse Model of Breast Cancer with Radiotherapy

Female Balb/c mice aged 8–10 weeks old were from Charles River (Kingston, ON, Canada) and were housed according to guidelines of the Canadian Council on Animal Care as approved by the University of Alberta/Cross Cancer Institute Animal Welfare Committees (AUP00000226 and AC16223). Mice were maintained at 21 ± 2 °C, 55% ± 5% humidity and a standard 12-h light-dark cycle. Mice had free access to a standard laboratory diet (4% fat) and water.

A syngeneic orthotopic model of breast cancer was employed using Balb/c mice that were injected into the 2nd left mammary fat pad with 20,000 4T1 breast cancer cells [34]. 4T1 tumors were allowed to grow for 12 days, at which point tumor sizes reached about 3 × 3 to 4 × 4 mm. Both normal Balb/c mice and tumor-bearing mice were irradiated using our image-guided small-animal radiation research platform (SARRP) from Xstrahl (Xstrahl Inc., Camberley, UK). Fractionated RT involved delivering 3 daily doses of 7.5 Gy of 220 kVp X-rays using 2 intersecting beams at angles of between 45° and 65° and a 10 mm square collimator with the isocenter positioned at the center of the tumor or fat pad. RT dose treatment plans were developed based on cone beam computed tomography images measured with the SARRP and the integrated MuriPlan software after contouring the tumor shape and defining the isocenter. Mice were anesthetized with isoflurane in 100% oxygen for each session. Tumor growth was monitored by two orthogonal caliper measurements and tumor volume was estimated from width^2^ × length/2. The mice were anesthetized terminally with intraperitoneal injection of 50 mg/kg sodium pentobarbital, and blood was collected by cardiac puncture using an EDTA coated syringe. Tumors and adipose tissues from the area adjacent to the tumor, as well as from fat pads of control (tumor-free) mice, were collected and frozen at −80 °C until analysis. Samples of the tumors and adipose tissues were also fixed with 10% formalin for immunohistochemistry.

### 4.3. Adipose Tissue Culture and Radiation Exposure

Human samples were obtained with approval of the University of Alberta Health Research Ethics Board ID Pro00018758 with written, informed consent. Human breast adipose tissue was obtained from breast reduction surgery and neck adipose tissue was obtained during surgeries for thyroid disease. Adipose tissues were handled as described previously, and the site of adipose tissue collection did not affect the results [58]. For γ-radiation exposure, the adipose tissue was incubated overnight and then exposed to 1 Gy of γ-radiation at a dose rate of 1.18 Gy/min using a ^60^Co GammaCell Irradiator (Atomic Energy of Canada Limited, Chalk River, ON, Canada).

### 4.4. Multiplex Analysis of Cytokines and Hormones

Mouse plasma and tissue homogenates were analyzed for cytokines/chemokines and hormones using Mouse Cytokine/Chemokine 32-plex ELISA and Mouse Metabolic 11-plex arrays by Eve Technologies Corp. (Calgary, AB, Canada). Concentrations obtained from the assay were normalized to both the volume of medium and protein concentration of the samples as determined by the BCA protein assay (Thermo Fisher Scientific, Rockford, IL, USA).

### 4.5. Measurement of ATX Activity

ATX activity was measured in culture medium or in mouse plasma and tissues as described previously based on choline release from lysophosphatidylcholine [61]. For detecting ATX activity in the adipose tissue, tissue homogenates were mixed with the same volume of 8 mM LPC-C14:0 in buffer A (100 mM Tris-HCl, pH 9.0; 500 mM NaCl; 5 mM MgCl_2_; and 0.05% v/v Triton X-100) and incubated for 3 h at 37 °C. For detecting ATX activity in plasma containing EDTA as an anticoagulant, the assay buffer A was supplemented with 25 mM CaCl_2_ and 25 mM MgCl_2_. After the incubation, 20-μL samples were mixed with 90 μL of buffer C containing 88 μL buffer B (100 mM Tris-HCl, pH 8.5 and 5 mM CaCl_2_), 0.7 μL of 10 mM Amplex^TM^ Red, 0.1 μL of 1000 U/mL horseradish peroxidase and 1.2 μL of 300 U/mL choline oxidase. Choline formation was measured at λ_Ex_ = 544 nm/λ_Em_ = 590 nm using a Fluoroskan Ascent™ FL Fluorometer (Thermo Fisher Scientific, Vantaa, Finland).

### 4.6. Measurement of Plasma LPA Concentrations

Plasma LPA concentrations were measured as described previously [61]. Lysophospholipids were measured by liquid chromatography/tandem mass spectrometry with electrospray ionization in the negative ion mode using an Agilent 1200 series LC system coupled to a 3200 QTRAP mass spectrometer (AB Sciex, Concord, ON, Canada). The absolute amounts of C16:0-LPA, C18:0-LPA, C18:1-LPA and C20:4-LPA were determined from calibration curves using authentic standards. Relative levels of LPA-18:2, LPA 22:6, S1P and SA1P were compared between treatment and control samples based on the ratios of analytes to their internal standard peak areas.

### 4.7. Quantitative Real-Time PCR (qRT-PCR)

RNA was isolated using Trizol^®^ (Invitrogen Life Technologies, Carlsbad, CA, USA) with the Direct-zol™ RNA MiniPrep kit (Zymo Research, Irvine, CA, USA) according to the manufacturer’s instructions. Real-time PCR was then performed using primers, as described previously [58]. Quantitative RT-PCR analysis was performed using RT2 SYBRgreen quantitative PCR mastermix (Qiagen) in the Applied Biosystems 7500 real-time RT-PCR system (Life Technologies, Grand Island, NY, USA). Primers for Nrf2 were F: 5′-GGTTGGCCCTTTCCTGCTTT-3′ and R: 5′-TCCATGTCCCTTGACAGCACA-3′; primers for HO-1 were F: 5′-AACTTTCAGAAGGGCCAGGT-3′ and R: 5′-CTGGGCTCTCCTTGTTGC-3′; primers for ABCC1 were F: 5′-GTGGCTATCAAGGGCTCCGT-3′ and R: 5′-CCCACTGGGCAGGATTTCCA-3′; primers for ABCG2 were F: 5′-AGATGGGTTTCCAAGCGTTCAT-3′ and R: 5′-CCAGTCCCAGTACGACTGTGACA-3′; primers for NQO1 were F: 5′-TGAAGGACCCTGCGAACTTTC-3′, and R: 5′-GAACACTCGCTCAAACCAGC-3′. Relative abundance of target mRNA was determined from Ct values and normalized to the geometric mean of the housekeeping gene GAPDH.

### 4.8. Immunohistochemistry and Western Blots

Tumors and adipose tissues were fixed with 10% formalin followed by paraffin embedding and sectioning. Sample treatment and immuno-staining were performed according to the standard procedure. Antigen retrieval was performed by heating slides with 10 mM citric acid (pH 6.0) in a pressure cooker for 20 min. Images were acquired using a Zeiss Axioskop 2 imaging system (Carl Zeiss Canada, Toronto, ON, Canada). The average counts in 5 fields were calculated for each sample. Protein levels were measured by western blot as described previously [58]. Immunoblots were analyzed by the LI-COR Odyssey Imaging System (LI-COR Biosciences, Lincoln, NE, USA).

### 4.9. Statistics

Results were expressed as means ± SEMs. Statistical significance between paired groups was determined using paired *t*-tests. One or two-way ANOVAs with Bonferroni post hoc tests were used for multiple comparisons according to the experimental design using SPSS 16.0 software (SPSS Inc, Chicago, IL, USA). *p* < 0.05 was considered statistically significant.

## 5. Conclusions

The present work provides novel evidence that repeated fractions of RT to breast adipose tissue enhance inflammation through activation of the autotaxin–lysophosphatidate-inflammatory cycle. This wound healing response to RT-induced damage could decrease the efficacy of further fractions of RT.

## Supplementary Materials

The following are available online at https://www.mdpi.com/2072-6694/11/11/1816/s1. Figure S1: The original membranes as shown were cut according to the molecular weight markers and were then probed with the appropriate antibodies as shown in examples. Figure S2: Three 7.5 Gy fractions of RT did not change concentrations of LPA species, S1P and SA1P in tumors significantly.

## Abbreviations

ATX, autotaxin; COX-2, cyclooxygenase-2; Nrf2, nuclear erythroid 2-like 2; GCLC, glutamate-cysteine ligase catalytic subunit; HO-1, heme oxygenase-1; N-acetylcysteine, NAC; CCL, C-C motif chemokine ligand; CXCL, C-X-C motif chemokine ligand; G-CSF, granulocyte-colony stimulating factor; IL, interleukin; LPA, lysophosphatidic acid, which at physiological pH is in a salt form, lysophosphatidate; LPAR1,2, LPA receptors types 1- and 2; LPP, lipid phosphate phosphatase; RT, radiotherapy; SCF, stem cell factor; tBHQ, t-butyl-hydroxyquinone, TNFα, tumor necrosis factor-α; S1P, sphingosine 1-phosphate; SA1P, sphinganine 1-phosphate; VEGF, vascular endothelial growth factor.
