# Supplementary material for: Repeated Fractions of X-Radiation to the Breast Fat Pads of Mice Augment Activation of the Autotaxin-Lysophosphatidate-Inflammatory Cycle
Source: Cancers (Basel). 2019 Nov 19;11(11):1816. doi: 10.3390/cancers11111816 (PMC6895803; doi:10.3390/cancers11111816)
Supplement: Supplementary file 1 [file cancers-11-01816-s001.pdf]

Western blot Figure 3A

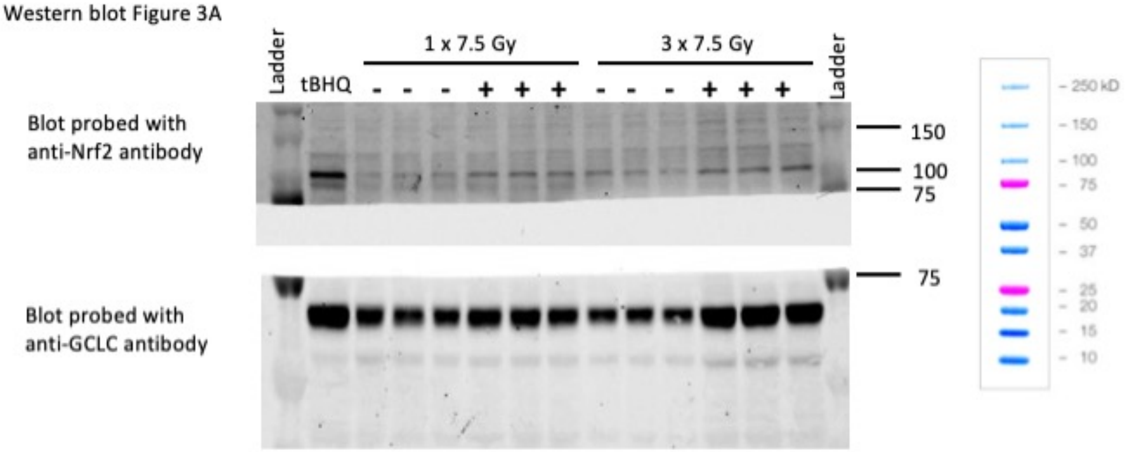

Western blot Figure 4

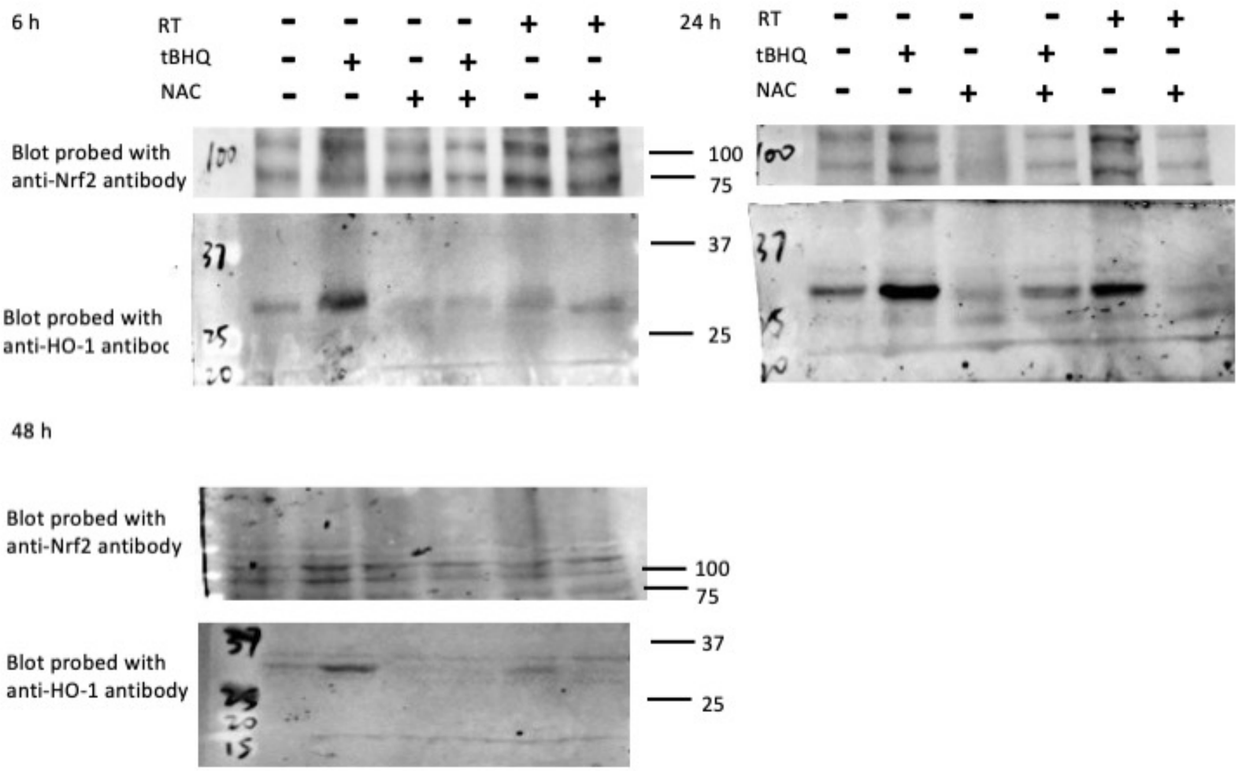

Western blot Figure 8C

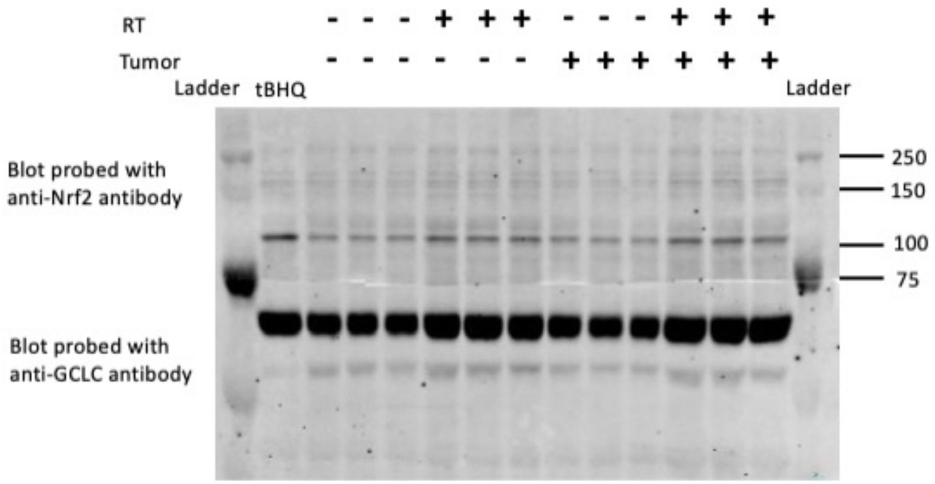

Western blot Figure 9B-a

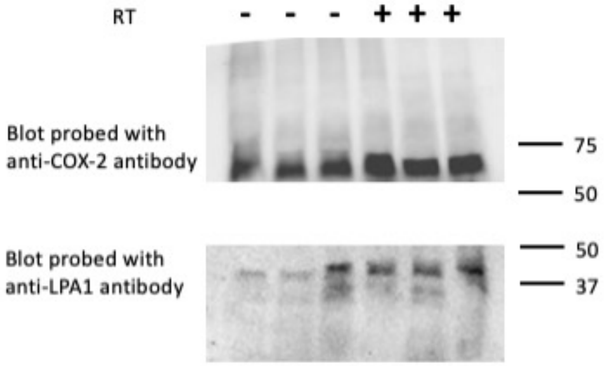

Supplementary Figure 1. The original membranes as shown were cut according to the molecular weight markers and were then probed with the appropriate antibodies as shown in examples.

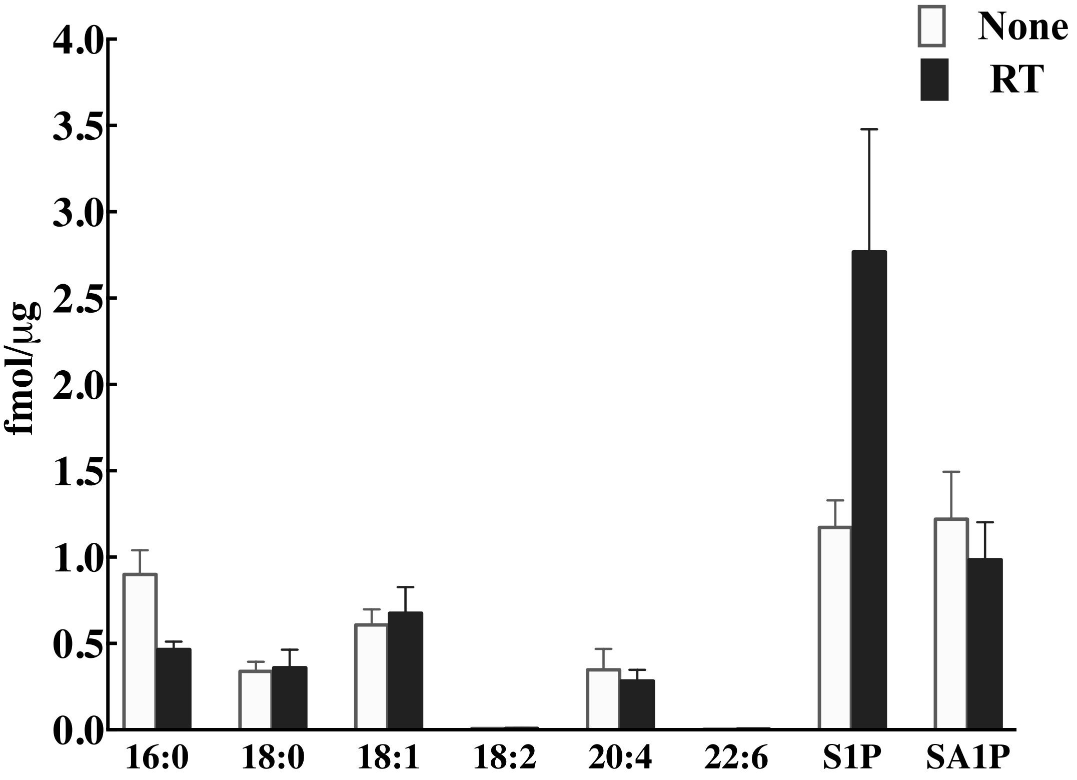

Supplementary Fig. 2. Three 7.5-Gy fractions of RT did not change the concentrations of LPA species, S1P and SA1P in tumors significantly. Mice with breast tumors were either untreated or were treated daily with X-rays for 3 days, and the concentrations of LPA species, S1P and SA1P in tumor tissues were measured at 48 h after the completion of RT. Absolute amounts of C16:0-LPA, C18:0-LPA, C18:1-LPA, C20:4-LPA were determined from the calibration curves, while LPA-18:2 LPA-22:6, S1P and SA1P were estimated from their peak areas relative to the isotopically-labelled standards. Results are expressed as means ± SEM with 6 mice/group.
